# Supplementary material for: The KASH5 protein involved in meiotic chromosomal movements is a novel dynein activating adaptor
Source: eLife. 2022 Jun 15;11:e78201. doi: 10.7554/eLife.78201 (PMC9242646; doi:10.7554/eLife.78201)

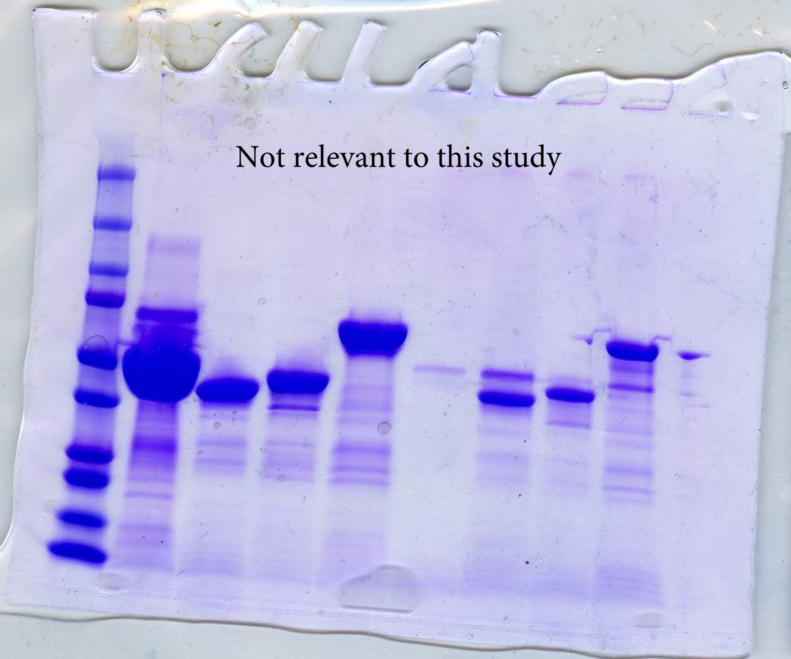
Not relevant to this study

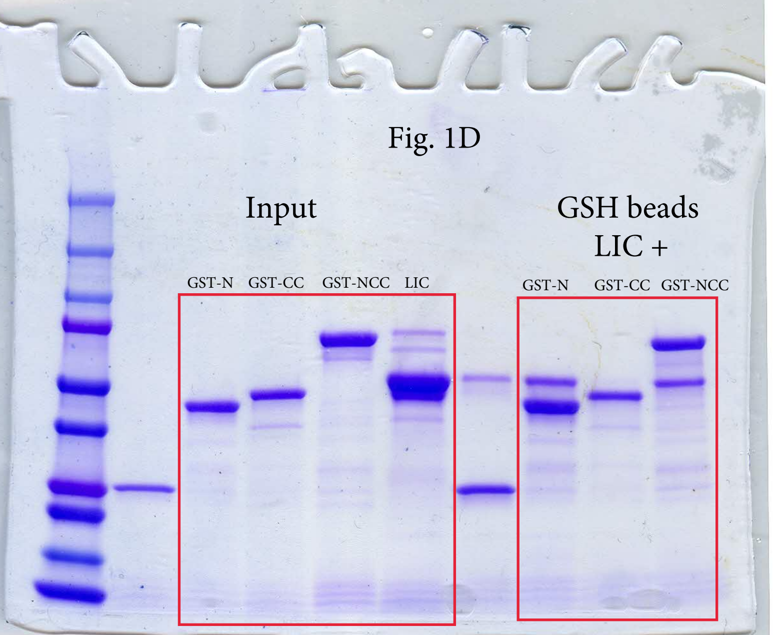
Fig. 1D
Input
GSH beads
LIC +
GST-N
GST-CC
GST-NCC
LIC
GST-N
GST-CC
GST-NCC

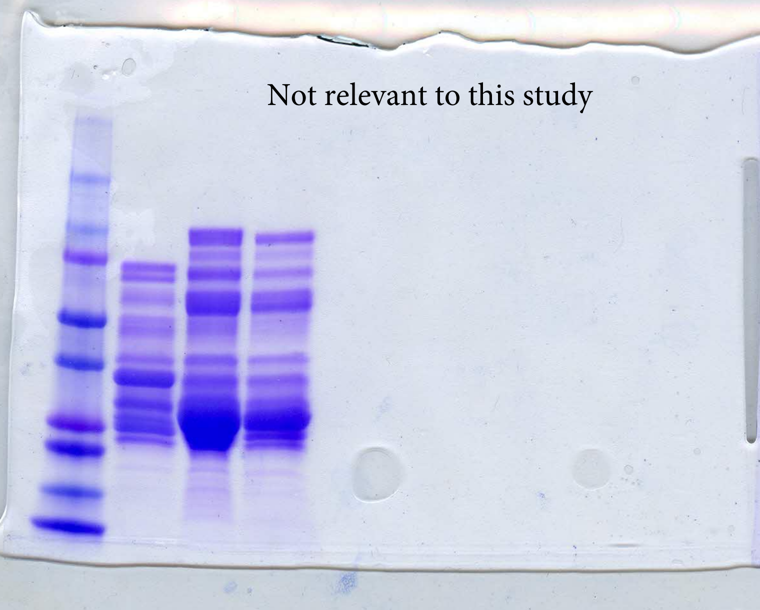
Not relevant to this study

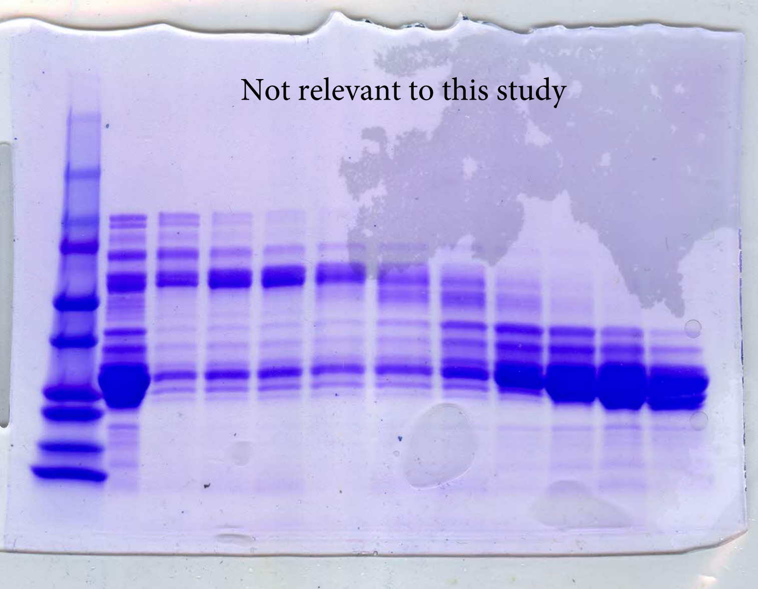
Not relevant to this study

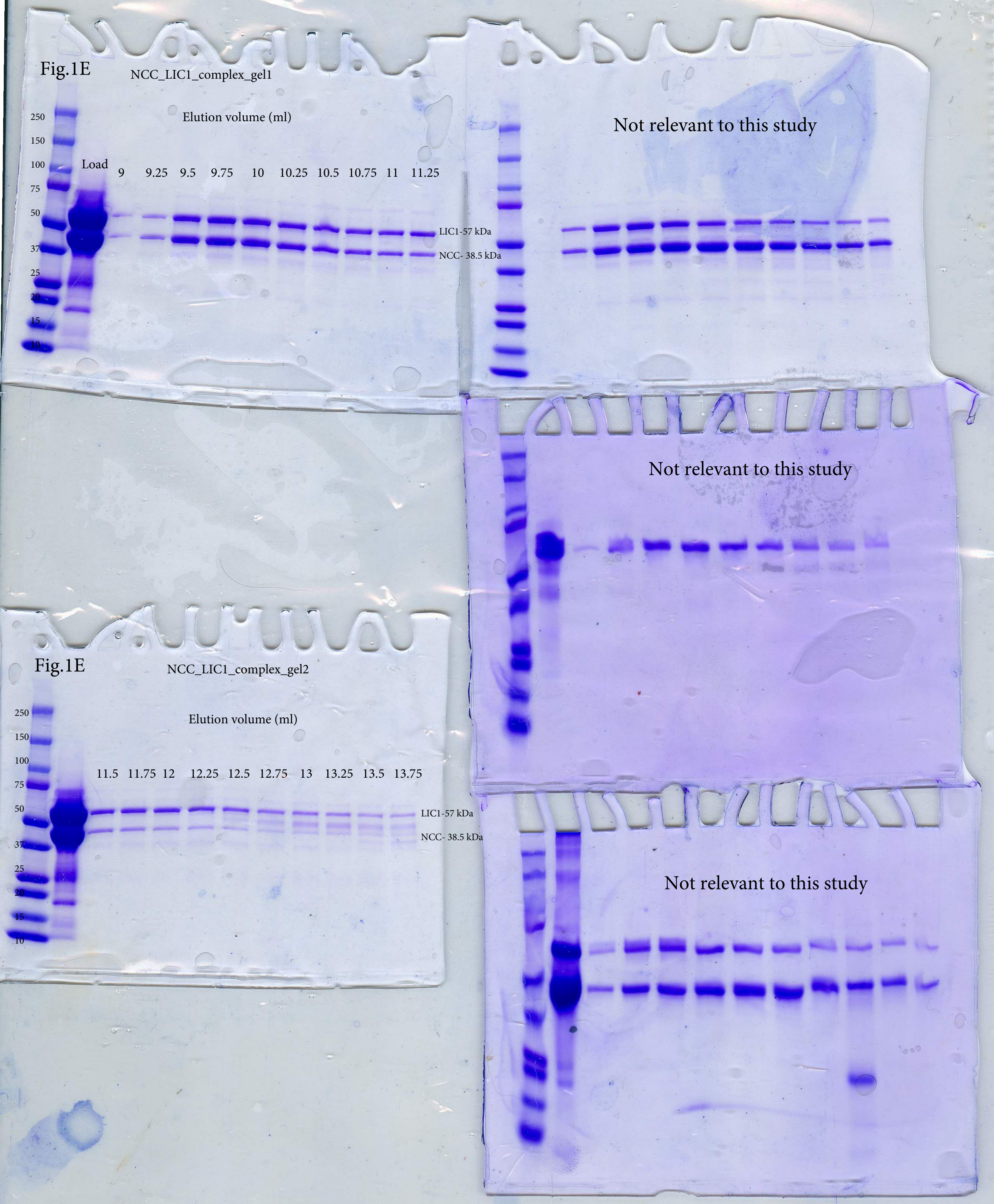
Fig.1E
NCC_LIC1_complex_gel1
Elution volume (ml)
250
150
100
75
50
37
25
20
15
10
Load
9
9.25
9.5
9.75
10
10.25
10.5
10.75
11
11.25
LIC1-57 kDa
NCC- 38.5 kDa
Not relevant to this study
Not relevant to this study
Fig.1E
NCC_LIC1_complex_gel2
Elution volume (ml)
250
150
100
75
50
37
25
20
15
10
11.5
11.75
12
12.25
12.5
12.75
13
13.25
13.5
13.75
LIC1-57 kDa
NCC- 38.5 kDa
Not relevant to this study

Fig.1F_ KASH5-NCC_LICF477AF478A_no complex

Gel1

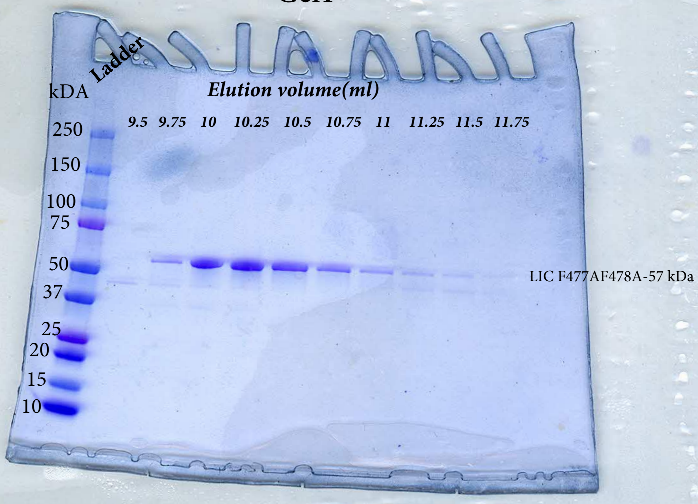

Gel2

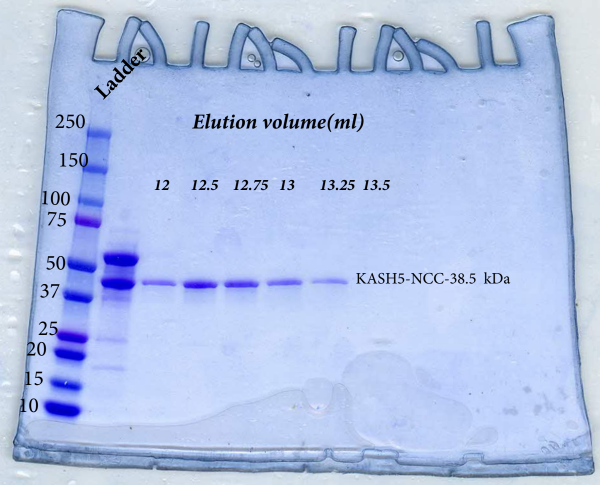

Supplement: Figure 1—source data 1. [file elife-78201-fig1-data1.pdf]
