# Supplementary material for: The KASH5 protein involved in meiotic chromosomal movements is a novel dynein activating adaptor
Source: eLife. 2022 Jun 15;11:e78201. doi: 10.7554/eLife.78201 (PMC9242646; doi:10.7554/eLife.78201)
Supplement: Figure 1—figure supplement 1—source data 1. [file elife-78201-fig1-figsupp1-data1.pdf]

White-HRP exposure merged images

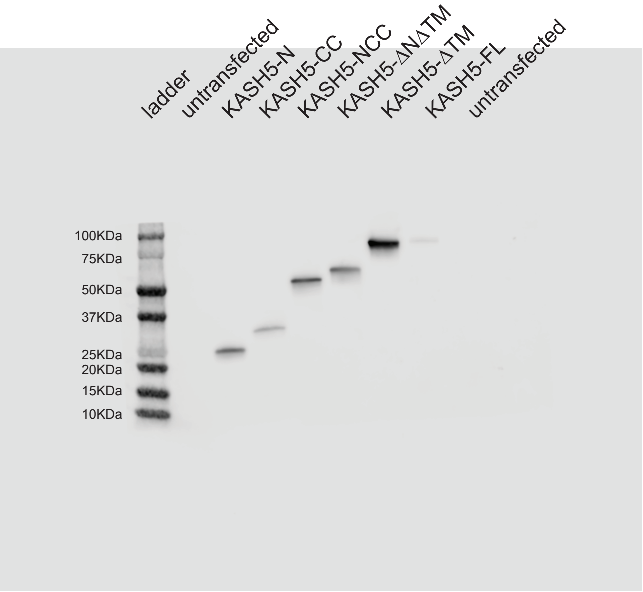

Input- anti FLAG

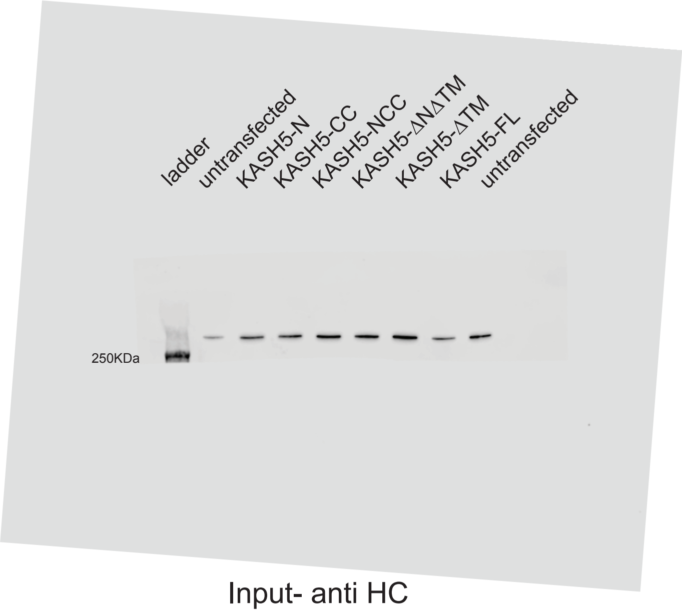

Input- anti HC

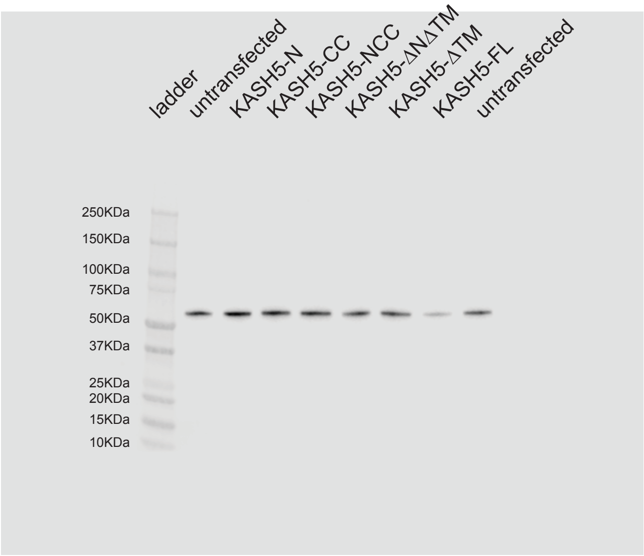

Input- anti LIC

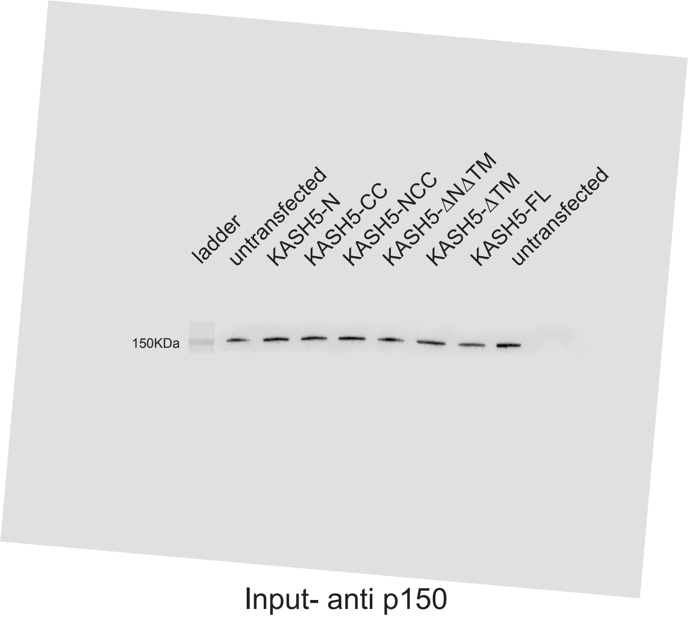

Input- anti p150

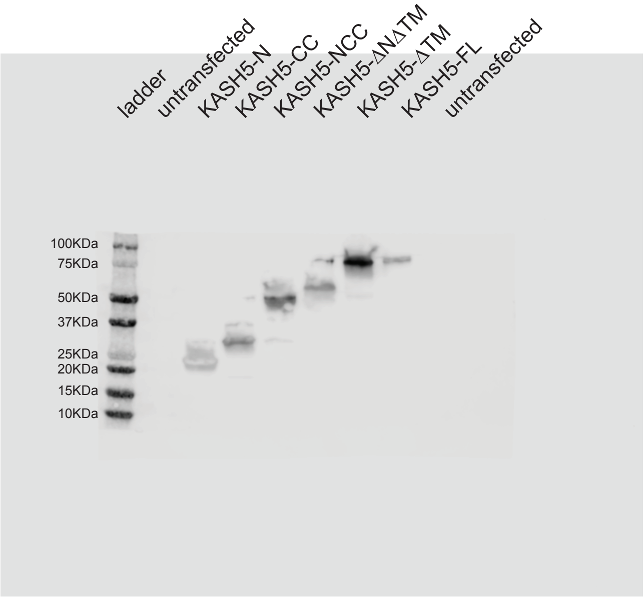

IP-anti FLAG

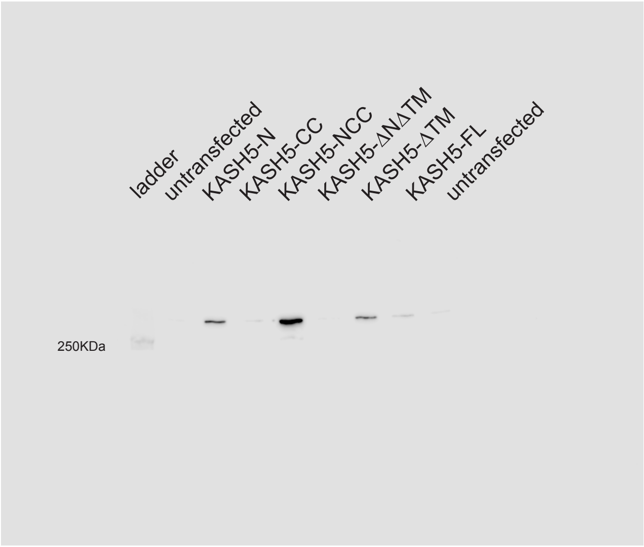

IP- anti HC

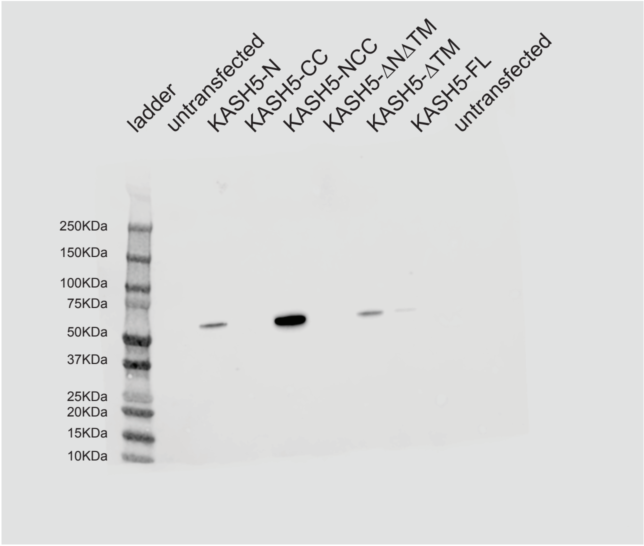

IP- anti LIC

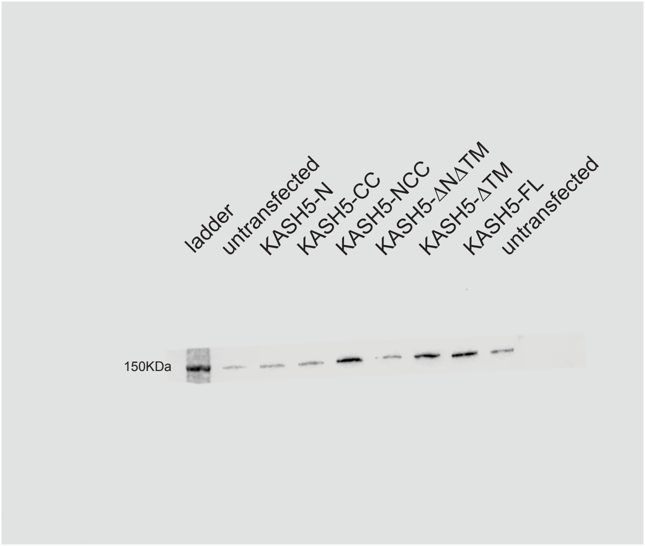

IP- anti p150
