# Supplementary figures and images for: The KASH5 protein involved in meiotic chromosomal movements is a novel dynein activating adaptor
Source: eLife. 2022 Jun 15;11:e78201. doi: 10.7554/eLife.78201 (PMC9242646; doi:10.7554/eLife.78201)

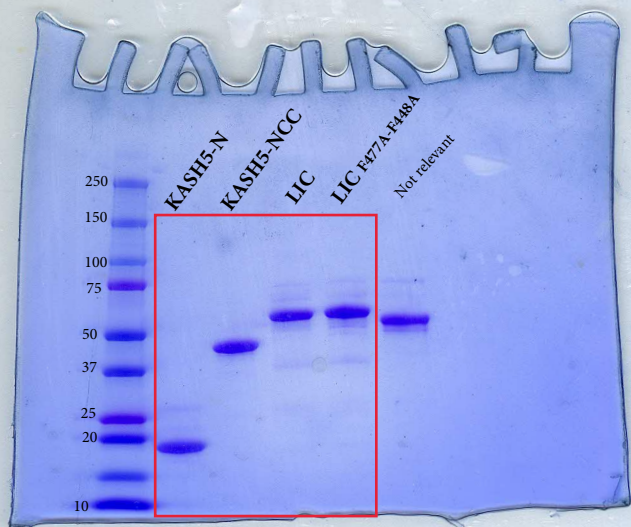

Supplement: Figure 1—figure supplement 1—source data 2. [file elife-78201-fig1-figsupp1-data2.pdf]

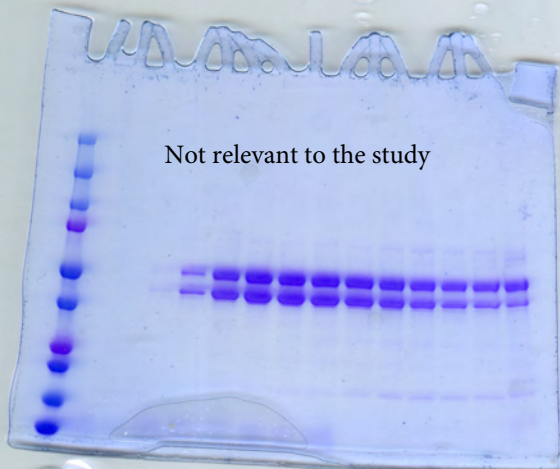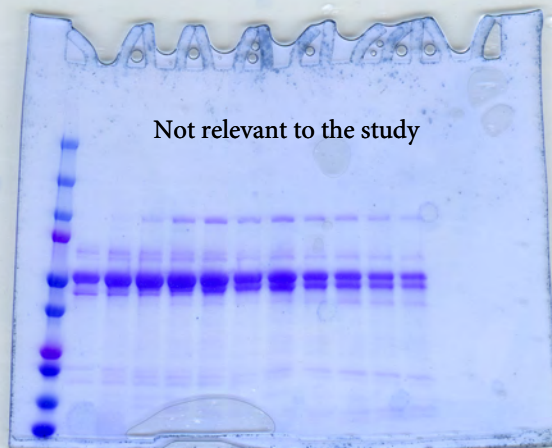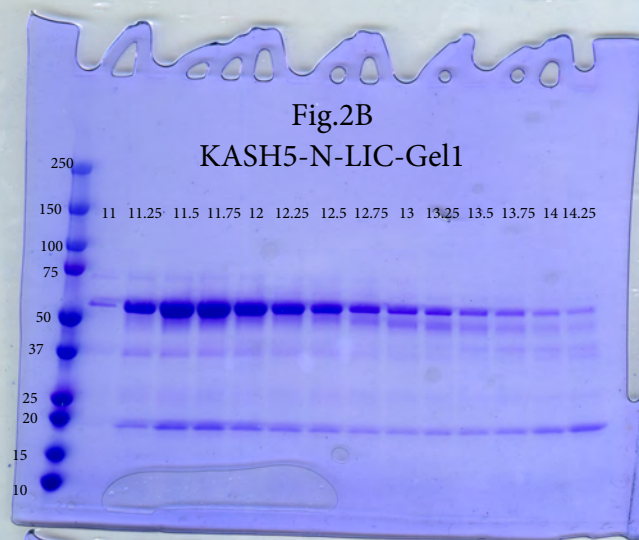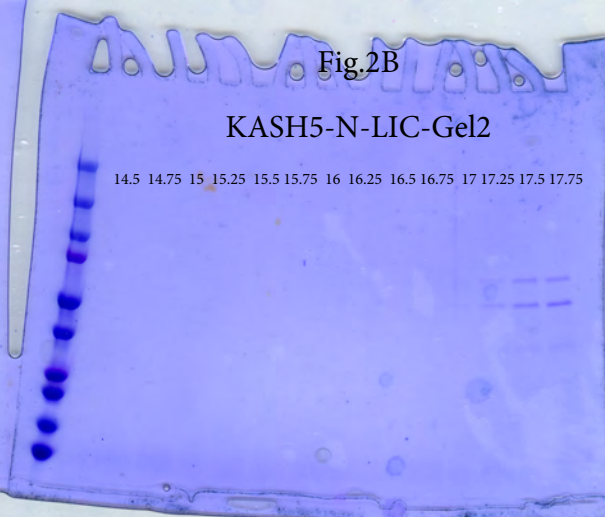

Elution volume(ml)

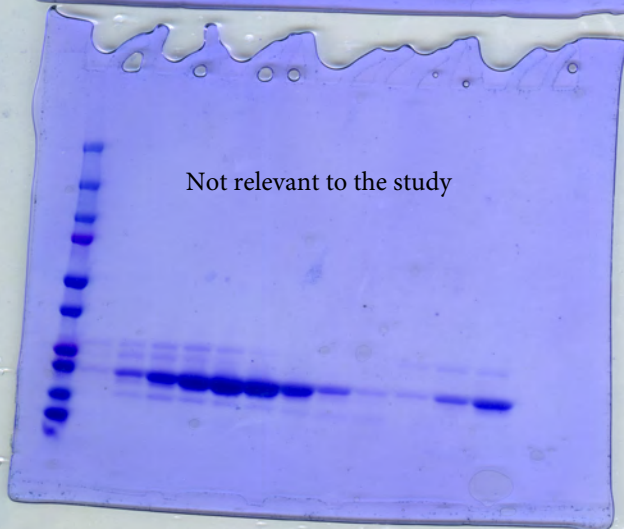

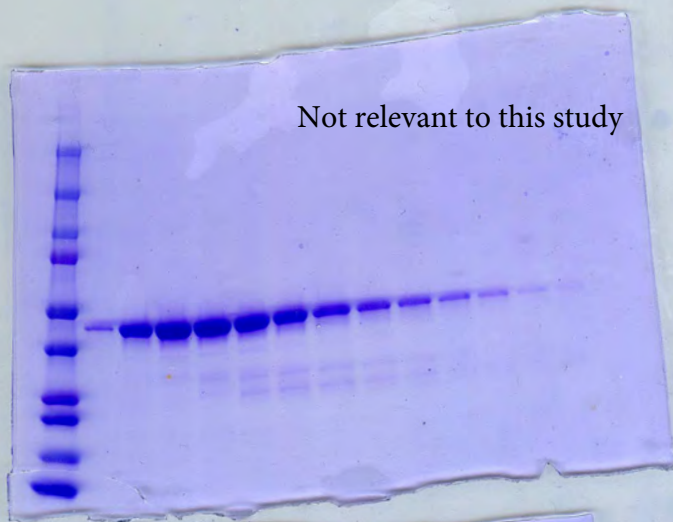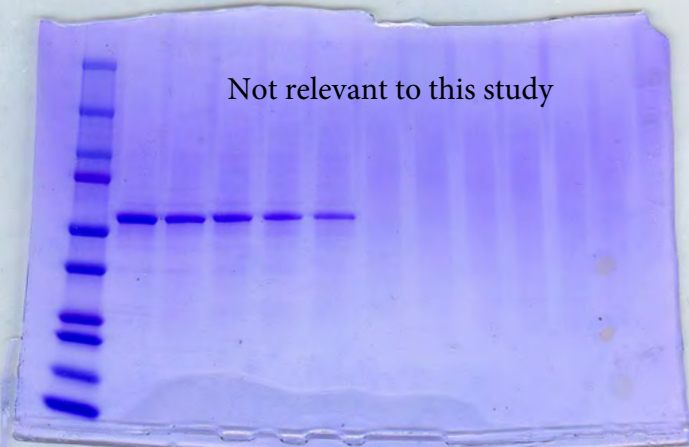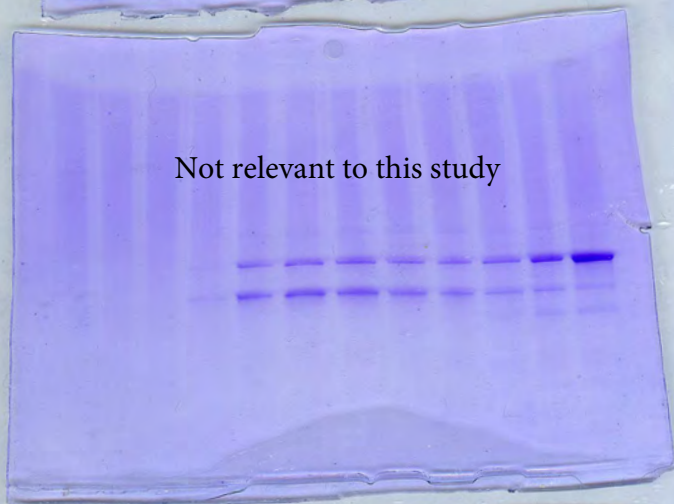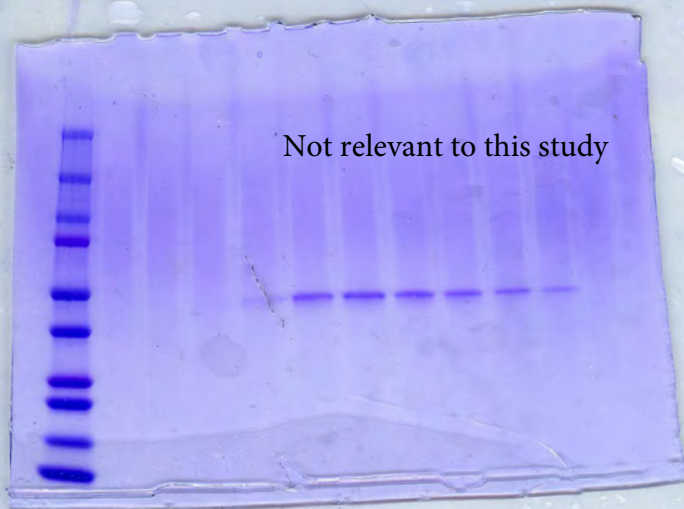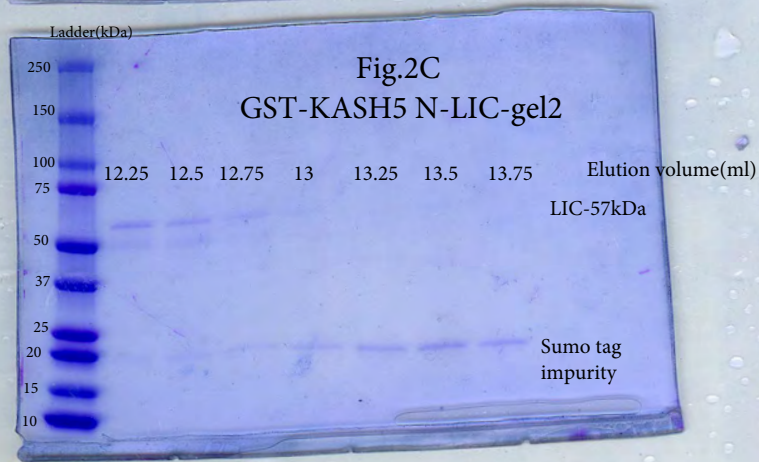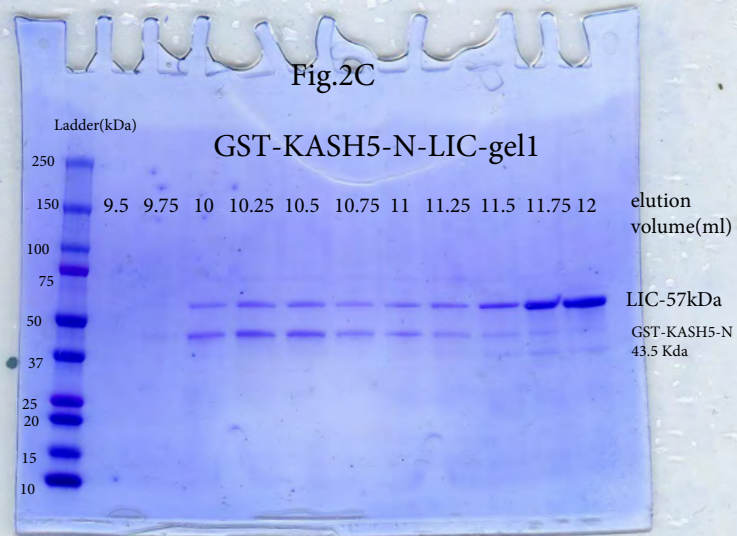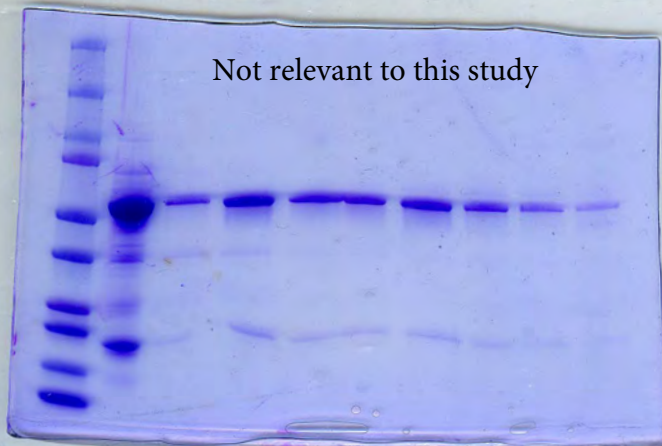

Supplement: Figure 2—source data 1. [file elife-78201-fig2-data1.pdf]

White-HRP exposure merged images

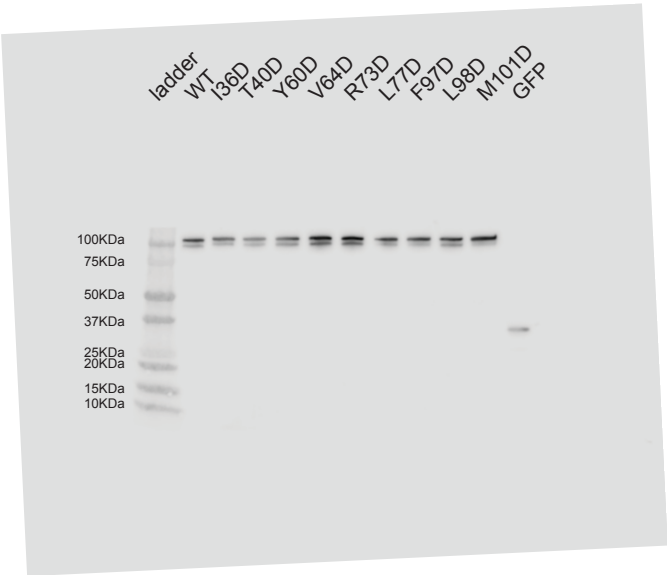

Input-anti FLAG

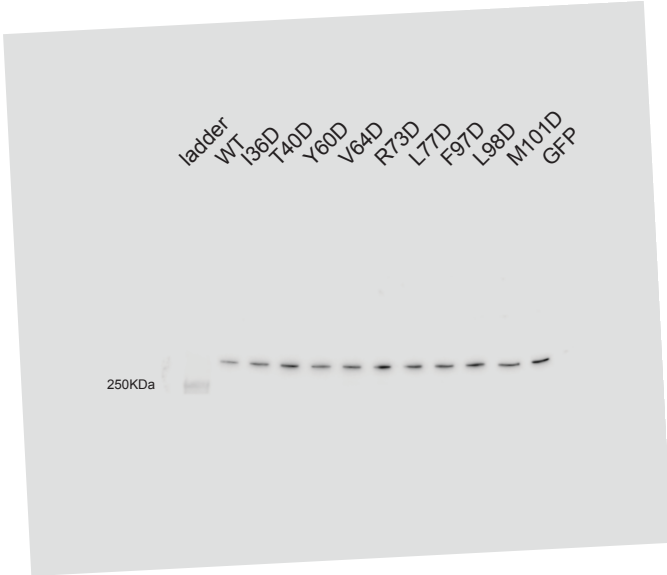

Input-anti HC

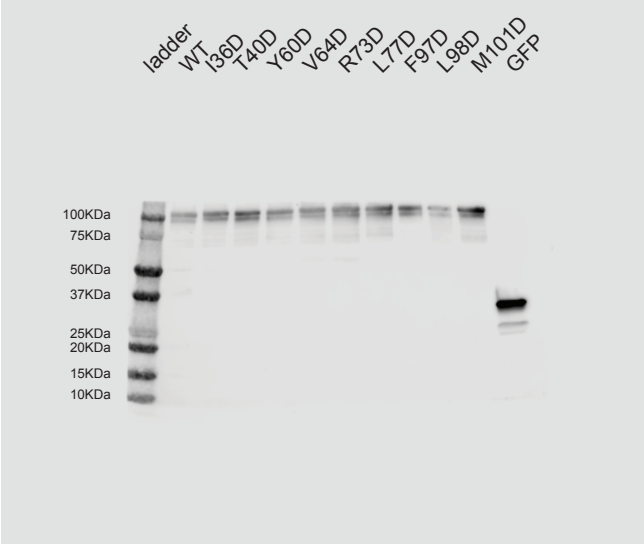

IP-anti FLAG

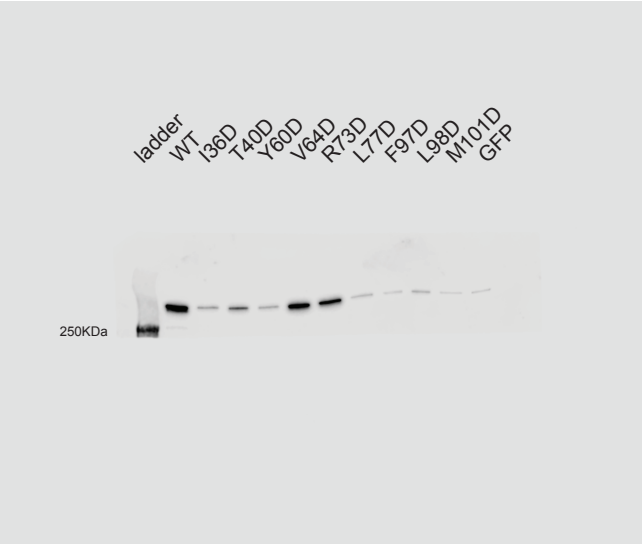

IP-anti HC

Supplement: Figure 4—source data 2. [file elife-78201-fig4-data2.pdf]

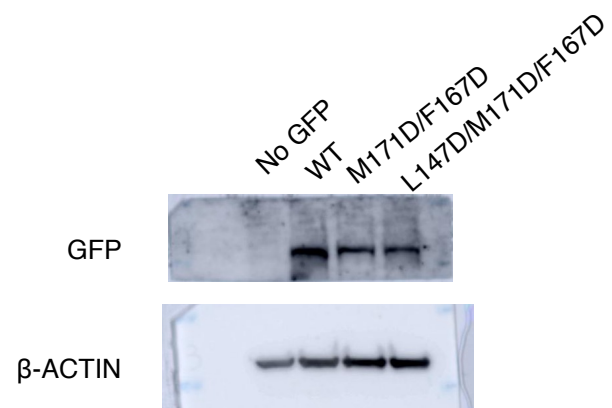

Supplement: Figure 6—figure supplement 1—source data 2. [file elife-78201-fig6-figsupp1-data2.pdf]
