# Supplementary material for: The KASH5 protein involved in meiotic chromosomal movements is a novel dynein activating adaptor
Source: eLife. 2022 Jun 15;11:e78201. doi: 10.7554/eLife.78201 (PMC9242646; doi:10.7554/eLife.78201)
Supplement: Figure 3—figure supplement 1—source data 2. [file elife-78201-fig3-figsupp1-data2.pdf]

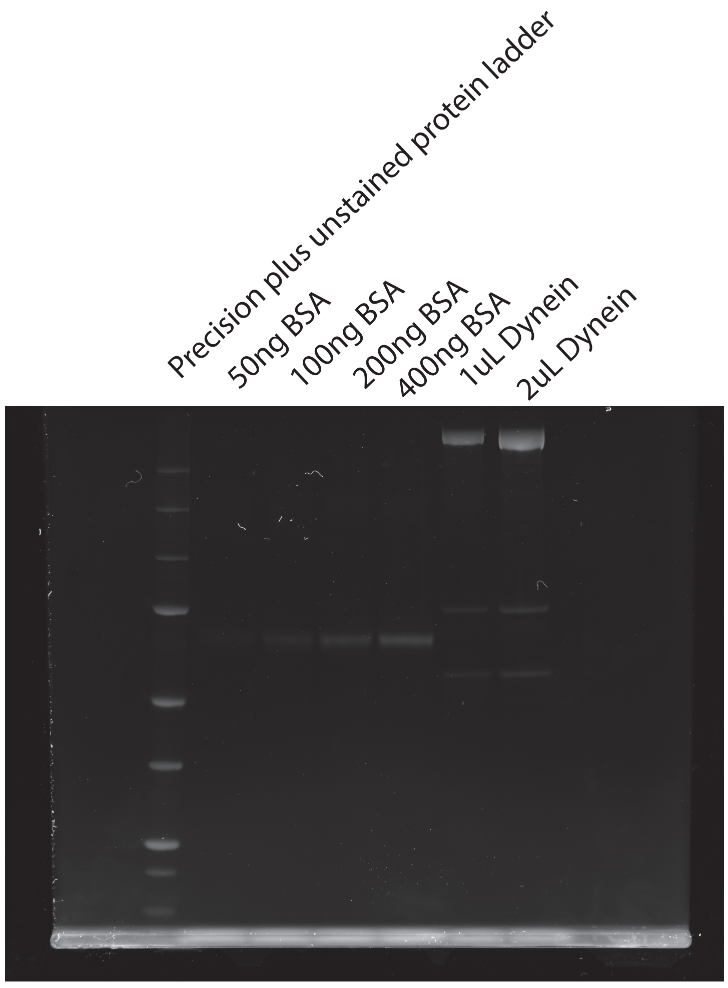
Precision plus unstained protein ladder
50ng BSA
100ng BSA
200ng BSA
400ng BSA
1uL Dynein
2uL Dynein

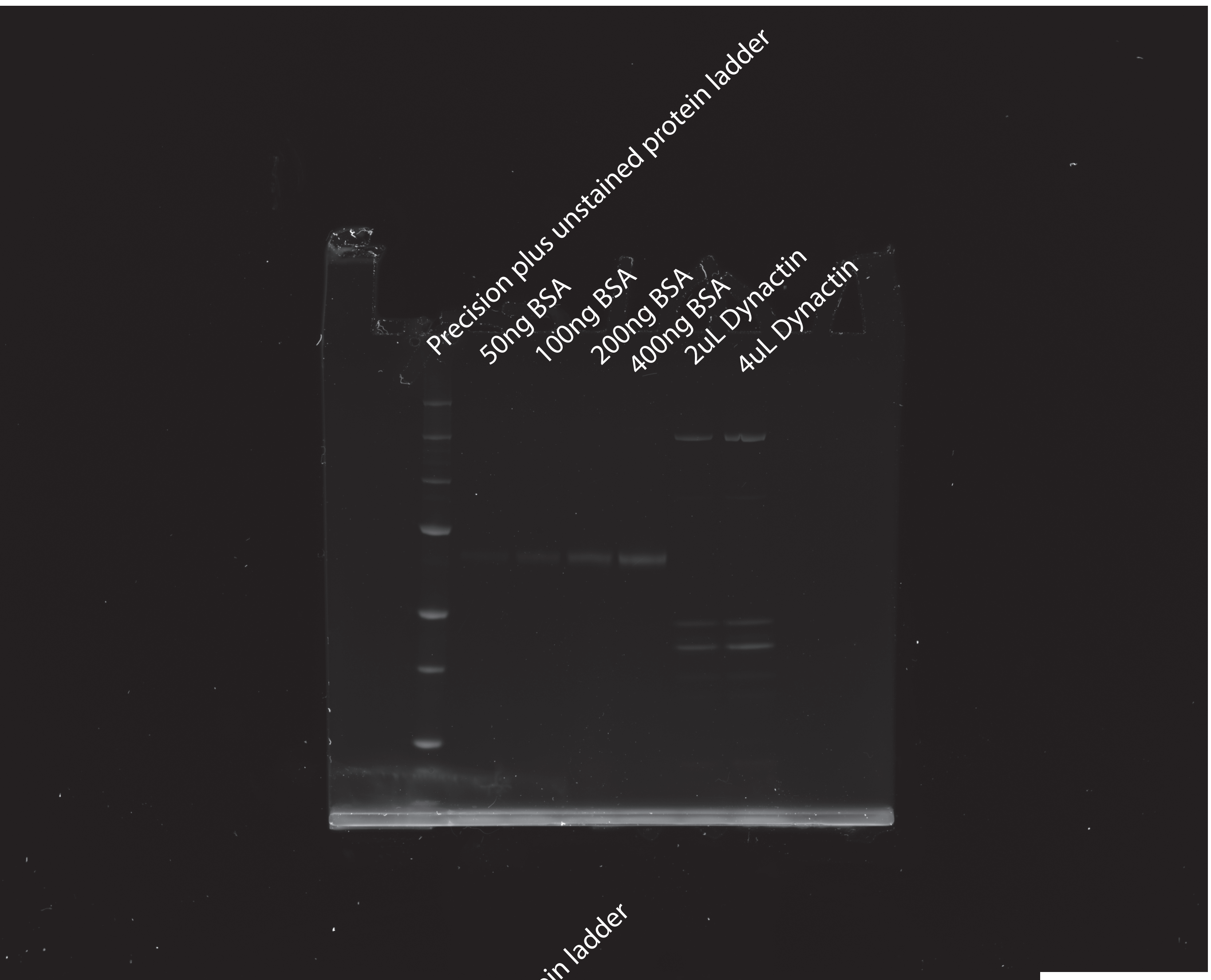
Precision plus unstained protein ladder
50ng BSA
100ng BSA
200ng BSA
400ng BSA
2uL Dynactin
4uL Dynactin

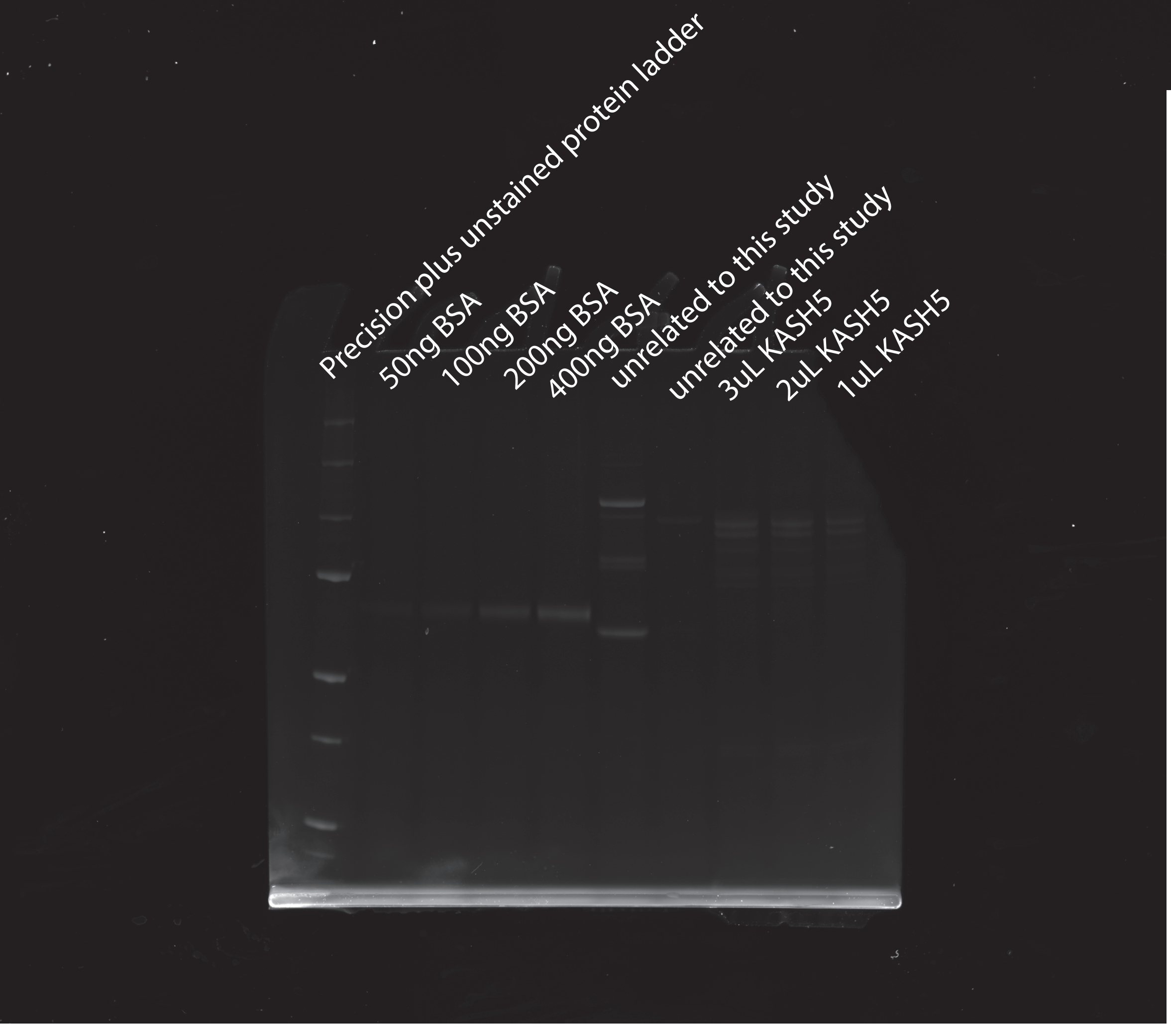
Precision plus unstained protein ladder
50ng BSA
100ng BSA
200ng BSA
400ng BSA
unrelated to this study
unrelated to this study
3uL KASH5
2uL KASH5
1uL KASH5
