# Supplementary material for: The KASH5 protein involved in meiotic chromosomal movements is a novel dynein activating adaptor
Source: eLife. 2022 Jun 15;11:e78201. doi: 10.7554/eLife.78201 (PMC9242646; doi:10.7554/eLife.78201)
Supplement: Figure 6—source data 2. [file elife-78201-fig6-data2.pdf]

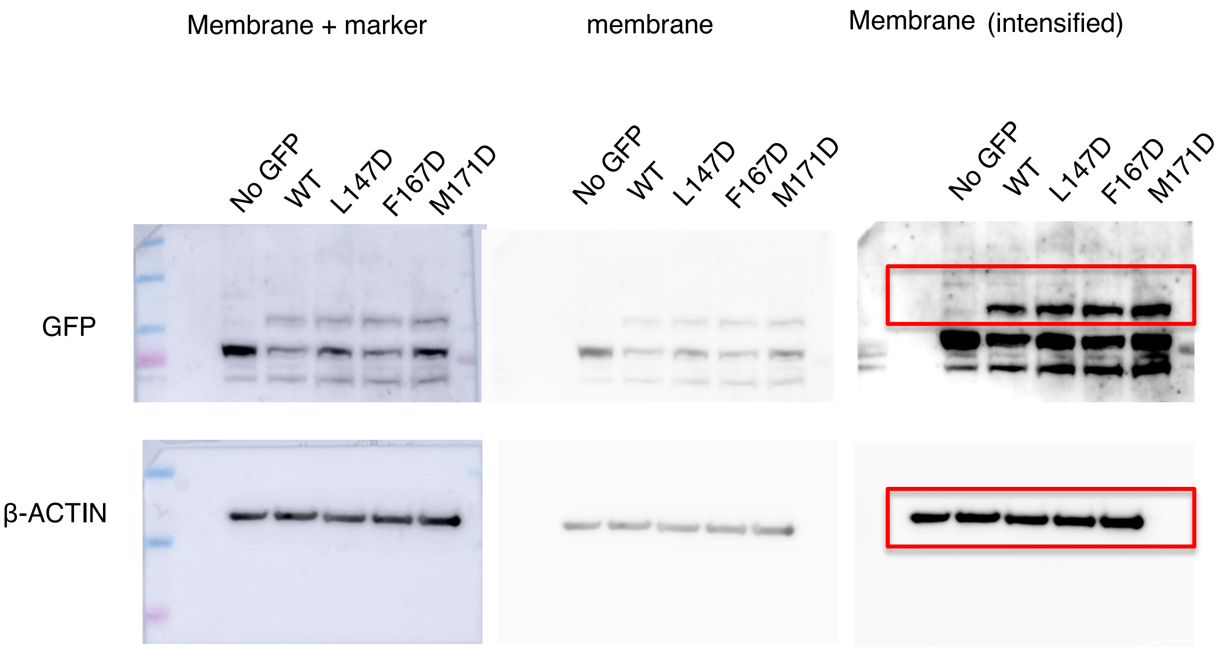

Red box indicates region shown in figure
