# Supplementary material for: The KASH5 protein involved in meiotic chromosomal movements is a novel dynein activating adaptor
Source: eLife. 2022 Jun 15;11:e78201. doi: 10.7554/eLife.78201 (PMC9242646; doi:10.7554/eLife.78201)
Supplement: Supplementary file 1. — This table contains all Kd values determined via isothermal titration calorimetry (ITC); all median velocity, mean percent processivity, median run length, and mean landing rates determined via total internal reflection fluorescence (TIRF); all median intensities determined via immunofluorescence; and reports all statistical tests used and all p-values determined in the manuscript. [file elife-78201-supp1.docx]

**Supplemental Table 1**

| **ISOTHERMAL CALORIMETRY** | | |
| --- | --- | --- |
| **Figure** | **Sample Name** | **Kd** |
| Fig. 2 | LIC_433-458_ titrated in KASH5-NCC | 4.3 µM ± 0.1 |
| Fig.2SC | LIC_433-458_ titrated in KASH5-NCC with 5mM CaCl_2_ | 5.34 µM ±1.03 |
| Fig.2SD | LIC_433-458_ titrated in KASH5-NCC with 5mM EGTA | 4.6 µM ±  1.31 |

| **IP-MOTILITY TIRF** | | |
| --- | --- | --- |
| **Figure** | **Sample Name** | **Median Velocity** |
| Fig.3C | BicD2 | 0.577 |
| Fig.3C | KASH5-ΔTM | 0.538 |
| Fig.3C | KASH5-NCC | 0.506 |
| Fig.4H | KASH5-ΔTM ^I36D^ | 0.62 |
| Fig.4H | KASH5-ΔTM ^T40D^ | 0.613 |
| Fig.4H | KASH5-ΔTM ^Y60D^ | 0.54 |
| Fig.4H | KASH5-ΔTM ^V64D^ | 0.664 |
| Fig.4H | KASH5-ΔTM ^R73D^ | 0.575 |
| Fig.4H | KASH5-ΔTM ^L77D^ | 0.644 |
| Fig.4H | KASH5-ΔTM ^F97D^ | 0.50 |
| Fig.4H | KASH5-ΔTM ^L98D^ | 0.59 |
| Fig.4H | KASH5-ΔTM ^M101^ | 0.602 |
| Fig.4H | WT KASH5-ΔTM | 0.614 |
|  | | |
| **Figure** | **Sample Name** | **Mean % Processive events** |
| Fig.3SB | BicD2 | 88.681 |
| Fig.S3B | KASH5-ΔTM | 72.9247 |
| Fig.S3B | KASH5-NCC | 63.928 |
| Fig.4G | KASH5-ΔTM ^I36D^ | 72.8626 |
| Fig.4G | KASH5-ΔTM ^T40D^ | 69.3661 |
| Fig.4G | KASH5-ΔTM ^Y60D^ | 65.0247 |
| Fig.4G | KASH5-ΔTM ^V64D^ | 61.1738 |
| Fig.4G | KASH5-ΔTM ^R73D^ | 57.2474 |
| Fig.4G | KASH5-ΔTM ^L77D^ | 20.446 |
| Fig.4G | KASH5-ΔTM ^F97D^ | 21.3325 |
| Fig.4G | KASH5-ΔTM ^L98D^ | 65.9845 |
| Fig.4G | KASH5-ΔTM ^M101^ | 15.3303 |
| Fig.4G | WT KASH5-ΔTM | 70.7164 |
|  |  |  |
| **Figure** | **Sample Name** | **Median Run lengths** |
| Fig.3S1A | BicD2 | 4.17016 |
| Fig.3S1A | KASH5-ΔTM | 3.23985 |
| Fig.3S1A | KASH5-NCC | 3.0188 |
|  |  |  |
| **Figure** | **Sample Name** | **Mean Landing Rate (events um^-1^ min^-1^)** |
| Fig. 3S1C | BicD2 | 0.655672 |
| Fig. 3S1C | KASH5-ΔTM | 0.341354 |
| Fig. 3S1C | KASH5-NCC | 0.191699 |
| Fig. 4SA | KASH5-ΔTM ^I36D^ | 0.2505 |
| Fig. 4SA | KASH5-ΔTM ^T40D^ | 0.3064 |
| Fig. 4SA | KASH5-ΔTM ^Y60D^ | 0.1011 |
| Fig. 4SA | KASH5-ΔTM ^V64D^ | 0.1926 |
| Fig. 4SA | KASH5-ΔTM ^R73D^ | 0.1274 |
| Fig. 4SA | KASH5-ΔTM ^L77D^ | 0.00971 |
| Fig. 4SA | KASH5-ΔTM ^F97D^ | 0.009694 |
| Fig. 4SA | KASH5-ΔTM ^L98D^ | 0.1842 |
| Fig. 4SA | KASH5-ΔTM ^M101^ | 0.005557 |
| Fig. 4SA | WT KASH5-ΔTM | 0.2268 |

| **Figure** | **Pairwise comparison** | **Velocity** | |
| --- | --- | --- | --- |
|  |  | **Summary** | **Adjusted p-value** |
| Fig.3C | BicD2 vs. KASH5_ΔTM | ns | 0.2008 |
| Fig.3C | BicD2 vs. KASH5_NCC | ** | 0.0018 |
| Fig.4H | WT KASH5-ΔTM vs. KASH5-ΔTM ^I36D^ | ns | >0.9999 |
| Fig.4H | WT KASH5-ΔTM vs. KASH5-ΔTM ^T40D^ | ns | >0.9999 |
| Fig.4H | WT KASH5-ΔTM vs. KASH5-ΔTM ^Y60D^ | ns | 0.1259 |
| Fig.4H | WT KASH5-ΔTM vs. KASH5-ΔTM ^V64D^ | ns | >0.9999 |
| Fig.4H | WT KASH5-ΔTM vs. KASH5-ΔTM ^R73D^ | ns | 0.3146 |
| Fig.4H | WT KASH5-ΔTM vs. KASH5-ΔTM ^L77D^ | ns | >0.9999 |
| Fig.4H | WT KASH5-ΔTM vs. KASH5-ΔTM ^F97D^ | ns | >0.9999 |
| Fig.4H | WT KASH5-ΔTM vs. KASH5-ΔTM ^L98D^ | ns | >0.9999 |
| Fig.4H | WT KASH5-ΔTM vs. KASH5-ΔTM ^M101^ | ns | >0.9999 |
|  |  |  | |
| **Figure** | **Pairwise comparison** | **% Processivity** | |
|  |  | **Summary** | **Adjusted p-value** |
| Fig.3S1B | BicD2 vs. KASH5-ΔTM | ** | 0.0045 |
| Fig.3S1B | BicD2 vs. KASH5-NCC | ** | 0.0023 |
| Fig.4G | KASH5-ΔTM vs. KASH5-NCC | ns | 0.1360 |
| Fig.4G | WT KASH5-ΔTM vs. KASH5-ΔTM ^I36D^ | ns | >0.9999 |
| Fig.4G | WT KASH5-ΔTM vs. KASH5-ΔTM ^T40D^ | ns | >0.9999 |
| Fig.4G | WT KASH5-ΔTM vs. KASH5-ΔTM ^Y60D^ | ns | 0.9642 |
| Fig.4G | WT KASH5-ΔTM vs. KASH5-ΔTM ^V64D^ | ns | 0.6785 |
| Fig.4G | WT KASH5-ΔTM vs. KASH5-ΔTM ^R73D^ | ns | 0.2625 |
| Fig.4G | WT KASH5-ΔTM vs. KASH5-ΔTM ^L77D^ | ** | 0.0014 |
| Fig.4G | WT KASH5-ΔTM vs. KASH5-ΔTM ^F97D^ | *** | 0.0007 |
| Fig.4G | WT KASH5-ΔTM vs. KASH5-ΔTM ^L98D^ | ns | 0.9998 |
| Fig.4G | WT KASH5-ΔTM vs. KASH5-ΔTM ^M101^ | *** | 0.0007 |
|  |  |  |  |
| **Figure** | **Pairwise comparison** | **Run lengths** | |
|  |  | **Summary** | **Adjusted p-value** |
| Fig. 3S1A | BicD2 vs. KASH5-ΔTM | **** | <0.0001 |
| Fig. 3S1A | BicD2 vs. KASH5-NCC | **** | <0.0001 |
| Fig.4SB | KASH5-ΔTM vs. KASH5-NCC | * | 0.0119 |
| Fig.4SB | WT KASH5-ΔTM vs. KASH5-ΔTM ^I36D^ | ns | >0.9999 |
| Fig.4SB | WT KASH5-ΔTM vs. KASH5-ΔTM ^T40D^ | ns | >0.9999 |
| Fig.4SB | WT KASH5-ΔTM vs. KASH5-ΔTM ^Y60D^ | **** | <0.0001 |
| Fig.4SB | WT KASH5-ΔTM vs. KASH5-ΔTM ^V64D^ | ns | >0.9999 |
| Fig.4SB | WT KASH5-ΔTM vs. KASH5-ΔTM ^R73D^ | ns | 0.0975 |
| Fig.4SB | WT KASH5-ΔTM vs. KASH5-ΔTM ^L77D^ | ns | >0.9999 |
| Fig.4SB | WT KASH5-ΔTM vs. KASH5-ΔTM ^F97D^ | ns | >0.9999 |
| Fig.4SB | WT KASH5-ΔTM vs. KASH5-ΔTM ^L98D^ | * | 0.0184 |
| Fig.4SB | WT KASH5-ΔTM vs. KASH5-ΔTM ^M101^ | ns | >0.9999 |
|  |  |  |  |
|  | **Pairwise comparison** | **Landing Rate** | |
|  |  | **Summary** | **Adjusted p-value** |
| Fig.3S1C | BicD2 vs. KASH5-ΔTM | ns | 0.0762 |
| Fig.3S1C | BicD2 vs. KASH5-NCC | ns | 0.2037 |
| Fig.3S1C | KASH5-ΔTM vs. KASH5-NCC | ns | 0.0945 |
| Fig.4SA | WT KASH5-ΔTM vs. KASH5-ΔTM ^I36D^ | ns | 0.9924 |
| Fig.4SA | WT KASH5-ΔTM vs. KASH5-ΔTM ^T40D^ | ns | 0.8824 |
| Fig.4SA | WT KASH5-ΔTM vs. KASH5-ΔTM ^Y60D^ | ns | 0.0507 |
| Fig.4SA | WT KASH5-ΔTM vs. KASH5-ΔTM ^V64D^ | ns | 0.965 |
| Fig.4SA | WT KASH5-ΔTM vs. KASH5-ΔTM ^R73D^ | ns | 0.1378 |
| Fig.4SA | WT KASH5-ΔTM vs. KASH5-ΔTM ^L77D^ | * | 0.0141 |
| Fig.4SA | WT KASH5-ΔTM vs. KASH5-ΔTM ^F97D^ | * | 0.0139 |
| Fig.4SA | WT KASH5-ΔTM vs. KASH5-ΔTM ^L98D^ | ns | 0.9893 |
| Fig.4SA | WT KASH5-ΔTM vs. KASH5-ΔTM ^M101^ | * | 0.0132 |

| **PURE PROTEIN TIRF** | | |
| --- | --- | --- |
| **Figure** | **Sample Name** | **Median Velocity** |
| Fig.3G | Dynein+Dynactin (DD) | 0.245 |
| Fig.3G | Dynein+Dynactin+Lis1 (DD+Lis1) | 0.396 |
| Fig.3G | Dynein+Dynactin+KASH5-**ΔTM**  (DDK) | 0.32 |
| Fig.3G | Dynein+Dynactin+KASH5-**ΔTM +** Lis1 (DDK+Lis1) | 0.577 |
|  |  |  |
| **Figure** | **Sample Name** | **Mean % processivity** |
| Fig.3S1F | Dynein+Dynactin (DD) | 5.939 |
| Fig.3S1F | Dynein+Dynactin+Lis1 (DD+Lis1) | 6.169 |
| Fig.3S1F | Dynein+Dynactin+KASH5-**ΔTM** (DDK) | 37.35 |
| Fig.3S1F | Dynein+Dynactin+KASH5-**ΔTM+**Lis1 (DDK+Lis1) | 51.76 |
|  |  |  |
| **Figure** | **Sample Name** | **Run Lengths** |
| Fig.3S1E | Dynein+Dynactin (DD) | 2.939 |
| Fig.3S1F | Dynein+Dynactin+Lis1 (DD+Lis1) | 3.672 |
| Fig.3S1F | Dynein+Dynactin+KASH5-**ΔTM** (DDK) | 3.562 |
|  | Dynein+Dynactin+KASH5-**ΔTM+**Lis1 (DDK+Lis1) | 4.431 |
| **Figure** | **Sample Name** | **Landing Rate (Runs/µM/nM/min)** |
| Fig.3H | Dynein+Dynactin (DD) | 0.042 |
| Fig.3H | Dynein+Dynactin+Lis1 (DD+Lis1) | 0.139 |
| Fig.3H | Dynein+Dynactin+KASH5-**ΔTM**  (DDK) | 0.53 |
| Fig.3H | Dynein+Dynactin+KASH5- **ΔTM** (DDK+Lis1) | 2.7 |

|  | **Pairwise comparison** | **Velocity** | |
| --- | --- | --- | --- |
|  |  | **Summary** | **Adjusted p-value** |
| Fig.3G | DDK vs. DD | ns | 0.2472 |
| Fig.3G | DDK vs. DDK + Lis1 | **** | <0.0001 |
| Fig.3G | DDK + Lis1 vs. DD + Lis1 | ** | 0.0014 |
|  |  |  |  |
| **Figure** | **Pairwise comparison** | **Mean % processivity** | |
|  |  | **Summary** | **Adjusted p-value** |
| Fig.3S1F | DDK vs. DDK + Lis1 | ns | 0.1737 |
| Fig.3S1F | DDK vs. DD | *** | 0.0006 |
| Fig.3S1F | DDK vs. DD + Lis1 | ** | 0.0015 |
| Fig.3S1F | DDK + Lis1 vs. DD | ** | 0.0011 |
| Fig.3S1F | DDK + Lis1 vs. DD + Lis1 | ** | 0.0010 |
| Fig.3S1F | \| DD vs. DD + Lis1 \| \| --- \| | ns | >0.9999 |
|  |  |  |  |
| **Figure** | **Pairwise comparison** | **Run lengths** | |
|  |  | **Summary** | **Adjusted p-value** |
| Fig.3S1E | DDK vs. DDK + Lis1 | ** | 0.0029 |
| Fig.3S1E | DDK vs. DD | ns | 0.5295 |
| Fig.3S1E | DDK vs. DD + Lis1 | ns | >0.9999 |
| Fig.3S1E | DDK + Lis1 vs. DD | **** | <0.0001 |
| Fig.3S1E | DDK + Lis1 vs. DD + Lis1 | ns | 0.0978 |
| Fig.3S1E | \| DD vs. DD + Lis1 \| \| --- \| | ns | 0.6355 |
|  |  |  |  |
|  | **Pairwise comparison** | **Landing Rate (Runs/µM/nM/min)** | |
|  |  | **Summary** | **Adjusted p-value** |
| Fig.3H | DDK vs. DD | **** | <0.0001 |
| Fig.3H | DDK vs. DDK + Lis1 | * | 0.0147 |
| Fig.3H | DDK + Lis1 vs. DD + Lis1 | ** | 0.0071 |

| **IMMUNOFLUORESCENCE** | | |
| --- | --- | --- |
|  | **Sample Name** | **Median of Dynein Intensity (Nuclear Envelope: Cytoplasm)** |
| Fig.5B | Untransfected | 1.409 |
| Fig.5B | SUN1 only | 1.264 |
| Fig.5B | KASH5- **ΔTM** | 1.356 |
| Fig.5B | KASH5-FL | 1.942 |
| Fig.5B | KASH5-FL^L77D^ | 1.487 |
| Fig.5B | KASH5-FL^F97D^ | 1.45 |
| Fig.5B | KASH5-FL^M101D^ | 1.688 |
|  |  |  |
|  | **Sample Name** | **Median of KASH5 intensity (Nuclear Envelope :Cytoplasm)** |
| Fig.5C | KASH5-**ΔTM** | 1.339 |
| Fig.5C | KASH5-FL | 2.116 |
| Fig.5C | KASH5-FL^L77D^ | 3.155 |
| Fig.5C | KASH5-FL^F97D^ | 2.461 |
| Fig.5C | KASH5-FL^M101D^ | 3.116 |

|  | **Pairwise comparison** | **Dynein Intensity (Nuclear Envelope :Cytoplasm)** | | |
| --- | --- | --- | --- | --- |
|  |  | **Summary** | | **Adjusted p-value** |
| Fig.5B | KASH5-FL vs. KASH5-FL^L77D^ | **** | | <0.0001 |
| Fig.5B | KASH5-FL vs. KASH5-FL^F97D^ | **** | | <0.0001 |
| Fig.5B | KASH5-FL vs. KASH5-FL^M101D^ | ns | | 0.1871 |
| Fig.5B | KASH5-FL vs. untransfected | **** | | <0.0001 |
| Fig.5B | KASH5-FL vs. KASH5-**ΔTM** | **** | | <0.0001 |
| Fig.5B | KASH5-FL vs. SUN1 only | **** | | <0.0001 |
| Fig.5B | KASH5-FL^L77D^ vs. KASH5-FL^F97D^ | ns | | >0.9999 |
| Fig.5B | KASH5-FL^L77D^ vs. KASH5-FL^M101D^ | ns | | 0.258 |
| Fig.5B | KASH5-FL^L77D^ vs. untransfected | ns | | >0.9999 |
| Fig.5B | KASH5-FL^L77D^ vs. KASH5-**ΔTM** | ns | | 0.9346 |
| Fig.5B | KASH5-FL^L77D^ vs. SUN1 only | * | | 0.0128 |
| Fig.5B | KASH5-FL^F97D^ vs. KASH5-FL^M101D^ | * | | 0.0106 |
| Fig.5B | KASH5-FL^F97D^ vs. untransfected | ns | | >0.9999 |
| Fig.5B | KASH5-FL^F97D^ vs. KASH5- **ΔTM** | ns | | >0.9999 |
| Fig.5B | KASH5-FL^F97D^ vs. SUN1 only | ns | | 0.0615 |
| Fig.5B | KASH5-FL^M101D^ vs. untransfected | ** | | 0.0015 |
| Fig.5B | KASH5-FL^M101D^ vs. KASH5-**ΔTM** | *** | | 0.0001 |
| Fig.5B | KASH5-FL^M101D^ vs. SUN1 only | **** | | <0.0001 |
| Fig.5B | untransfected vs. KASH5-**ΔTM** | ns | | >0.9999 |
| Fig.5B | untransfected vs. SUN1 only | ns | | 0.0706 |
| Fig.5B | KASH5- **ΔTM vs. SUN1 only** | ns | | >0.9999 |
|  |  |  | |  |
|  | **Pairwise comparison** | **KASH5 intensity (Nuclear Envelope :Cytoplasm)** | | |
|  |  | **Summary** | | **Adjusted p-value** |
| Fig.5C | KASH5-FL vs. KASH5-FL^L77D^ | **** | | <0.0001 |
| Fig.5C | KASH5-FL vs. KASH5-FL^F97D^ | ns | | 0.1664 |
| Fig.5C | KASH5-FL vs. KASH5-FL^M101D^ | **** | | <0.0001 |
| Fig.5C | KASH5-FL vs. KASH5-**ΔTM** | **** | | <0.0001 |
| Fig.5C | KASH5-FL^L77D^ vs. KASH5-FL^F97D^ | ** | | 0.0029 |
| Fig.5C | KASH5-FL^L77D^ vs. KASH5-FL^M101D^ | ns | | >0.9999 |
| Fig.5C | KASH5-FL^L77D^ vs. KASH5-**ΔTM** | **** | | <0.0001 |
| Fig.5C | KASH5-FL^F97D^ vs. KASH5-FL^M101D^ | *** | | 0.0004 |
| Fig.5C | KASH5-FL^F97D^ vs. KASH5-**ΔTM** | **** | | <0.0001 |
| Fig.5C | KASH5-FL^M101D^ vs. KASH5-**ΔTM** | **** | | <0.0001 |
|  |  |  | |  |
| **IMMUNOFLUORESCENCE** | | | | |
|  | **Sample Name** | **Relative p150 focus intensity (A.U.)** | | |
| Fig. 6B | No GFP | 1.000 | | |
| Fig. 6B | WT | 0.9044 | | |
| Fig. 6B | L147D | 0.7303 | | |
| Fig. 6B | F167D | 0.9017 | | |
| Fig. 6B | M171D | 0.9365 | | |
|  |  |  | | |
|  | **Sample Name** | **Relative GFP focus intensity (A.U.)** | | |
| Fig. 6C | WT | 1.000 | | |
| Fig. 6C | L147D | 0.9720 | | |
| Fig. 6C | F167D | 0.9876 | | |
| Fig. 6C | M171D | 0.9211 | | |
|  |  |  | | |
|  | **Sample Name** | **Relative p150 focus intensity (A.U.)** | | |
| Fig.6S1C | WT | 1.000 | | |
| Fig.6S1C | F167D/M171D | 1.037 | | |
| Fig.6S1C | L147D/F167D/M171D | 0.6441 | | |
|  |  |  | | |
|  | **Pairwise comparison** | **Relative p150 focus intensity (A.U.)** | | |
|  |  | **Summary** | **Adjusted p-value** | |
| Fig. 6B | No GFP vs. WT | ns | 0.1472 | |
| Fig. 6B | No GFP vs. L147D | **** | <0.0001 | |
| Fig. 6B | No GFP vs. F167D | ns | 0.1301 | |
| Fig. 6B | No GFP vs. M171D | ns | 0.4834 | |
|  |  |  |  | |
|  | **Pairwise comparison** | **Relative GFP focus intensity (A.U.)** | | |
|  |  | **Summary** | **Adjusted p-value** | |
| Fig. 6C | WT vs. L147D | ns | 0.9551 | |
| Fig. 6C | WT vs. F167D | ns | 0.9957 | |
| Fig. 6C | WT vs. M171D | ns | 0.5090 | |
|  |  |  |  | |
|  | **Pairwise comparison** | **Relative p150 focus intensity (A.U.)** | | |
|  |  | **Summary** | **Adjusted p-value** | |
| Fig.6S1C | WT vs F167D/M171D | ns | 0.8132 | |
| Fig.6S1C | WT vs L147D/F167D/M171D | **** | <0.0001 | |
